# Synthetic gene circuits for metabolic control: design trade-offs and constraints

**DOI:** 10.1098/rsif.2012.0671

**Published:** 2013-01-06

**Authors:** Diego A. Oyarzún, Guy-Bart V. Stan

**Affiliations:** Centre for Synthetic Biology and Innovation, Department of Bioengineering, Imperial College London, London SW7 2AZ, UK

**Keywords:** metabolic control, operon regulation, feedback control design

## Abstract

A grand challenge in synthetic biology is to push the design of biomolecular circuits from purely genetic constructs towards systems that interface different levels of the cellular machinery, including signalling networks and metabolic pathways. In this paper, we focus on a genetic circuit for feedback regulation of unbranched metabolic pathways. The objective of this feedback system is to dampen the effect of flux perturbations caused by changes in cellular demands or by engineered pathways consuming metabolic intermediates. We consider a mathematical model for a control circuit with an operon architecture, whereby the expression of all pathway enzymes is transcriptionally repressed by the metabolic product. We address the existence and stability of the steady state, the dynamic response of the network under perturbations, and their dependence on common tuneable knobs such as the promoter characteristic and ribosome binding site (RBS) strengths. Our analysis reveals trade-offs between the steady state of the enzymes and the intermediates, together with a separation principle between promoter and RBS design. We show that enzymatic saturation imposes limits on the parameter design space, which must be satisfied to prevent metabolite accumulation and guarantee the stability of the network. The use of promoters with a broad dynamic range and a small leaky expression enlarges the design space. Simulation results with realistic parameter values also suggest that the control circuit can effectively upregulate enzyme production to compensate flux perturbations.

## 1. Introduction

Synthetic biology aims at engineering cellular systems to perform customized and programmable biological functions. The seminal works published in 2000 [1,2] kick-started the development of a wide range of gene circuits with prescribed functions, including bacterial logic gates [3], mechanisms for programmed cell-to-cell communication [4] and light-responsive modules [5]. This progress has recently been followed by the so-called ‘second wave’ of synthetic biology [6], which aims at scaling up the designs from individual genetic modules to whole cellular systems that operate across different layers of cellular regulation, including signalling networks and metabolic pathways [7,8].

One of the most prominent applications of synthetic biology is the manipulation of bacterial metabolism for chemical production in sectors such as energy, biomedicine and food technology [6]. Effective control of metabolism hinges on the ability to upregulate or downregulate pathways in response to changes in the intracellular conditions, cell requirements or environmental perturbations [9]. These requirements call for dynamic control strategies that can modulate enzyme expression in a metabolite-dependent fashion [10,11]. One of the key bottlenecks in this respect is our limited understanding of how genetic design knobs modulate the metabolic responses.

The goal of this paper is to reveal new insights into the design limitations and trade-offs arising from the interplay between gene circuits and metabolic pathways. To that end, we analyse a dynamic model for a feedback system comprising nonlinear kinetic equations for the metabolic species, together with product-dependent enzyme expression controlled by a synthetic gene circuit. We focus on the existence and stability of the steady state, the dynamic response of the network under perturbations and the dependence of these on the design knobs of the synthetic gene circuit.

Two landmark implementations of engineered genetic–metabolic circuits are the genetic control of lycopene production [12] and the metabolic oscillator described in Fung *et al*. [13]. These works were followed up by the recent study by Zhang *et al*. [14], whereby the authors reported the first successful implementation of a genetic control circuit to increase biofuel production. In a way akin to man-made technological systems, the use of feedback control plays a pivotal role in ‘robustifying’ pathway dynamics under changing environmental conditions, cell-to-cell variability and biochemical noise. Despite the ubiquity of control engineering methods [15], only a few works have rigorously addressed the problem of genetic feedback design on the basis of mathematical models. Notably, Anesiadis *et al*. [16] demonstrated the use of a genetic toggle switch [2] as an ON–OFF controller for metabolism, whereas Dunlop *et al*. [17] explored different genetic control architectures for biofuel production.

**Figure 1.**
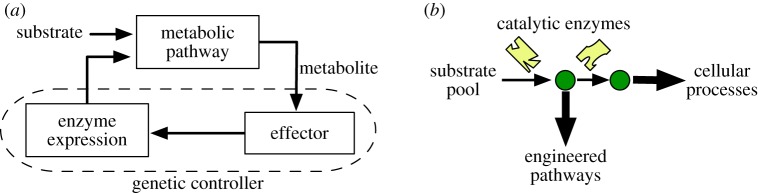
Control design for metabolic pathways. (*a*) Transcriptional regulation of metabolic pathways seen as a feedback control system: effector molecules (such as transcription factors) sense metabolite concentrations and modulate the expression of catalytic enzymes, which act as inputs to the pathway. (*b*) Engineered pathways can divert part of the native metabolic flux to the production of foreign compounds. (Online version in colour.)

From a control engineering standpoint, catalytic enzymes act as inputs to a metabolic pathway in order to drive the metabolite dynamics (i.e. the outputs). The pathway outputs are then sensed by metabolite-responsive molecules that can modulate enzyme expression levels (e.g. transcription factors (TFs) or riboswitches [18]). In the control engineering jargon, this feedback system can be seen as a ‘plant’ (i.e. the pathway to be controlled), and a ‘controller’ (i.e. the gene regulatory circuit controlling the expression of the catalytic enzymes); see figure 1*a*. The design of the genetic controller must then account for two complementary control objectives: firstly, it must dynamically adjust pathway activity to match the cellular demand for product and sustain the homeostatic balance of native cellular processes. Secondly, a common strategy in metabolic engineering is to modify host microbes by expressing heterologous enzymes that convert metabolic intermediates into a chemical of interest [19]. The consumption of intermediates diverts part of the flux allocated to the host native processes (figure 1*b*), and, therefore, the controller must also alleviate the impact of these engineered pathways on the native flux.

In this paper, we study an unbranched metabolic pathway under transcriptional repression from the product. The synthetic circuit consists of an operon encoding all the catalytic enzymes that is repressed by a product-responsive TF (§2). The operon feedback architecture mimics natural circuits enabling cellular adaptations to environmental perturbations (e.g. in bacterial amino acid metabolism [20] and nutrient uptake [21]). To maintain a general analysis, we do not specify the kinetics of the metabolic model, but rather work with a generic class of enzyme turnover rates satisfying mild assumptions. These are satisfied by a wide range of saturable enzyme kinetics, including Michaelis–Menten kinetics and cooperative behaviour described by sigmoidal kinetics [22]. We parameterize the genetic model in terms of the promoter characteristic and the ribosome binding site (RBS) strengths, which are typical design elements used as tuneable knobs in synthetic biology applications. As with the enzyme kinetics, we do not fix the shape of the promoter characteristic, but rather consider a generic class of repressive functions that account, in particular, for the standard Hill equation model for transcriptional repression [23].

Model analysis revealed that enzymatic saturation and promoter leaky expression limit the RBS strength design space (§3.1). These constraints must be satisfied to guarantee the existence of an equilibrium point, to prevent the accumulation of metabolites and to ensure the stability of the network under small perturbations. The feasible set for the RBS strengths depends critically on the promoter leakiness and substrate availability. Within the feasible set, RBS strengths may be used to fine-tune the balance between the intermediate metabolite levels and the gene expression burden imposed on the host cell. We also obtained analytical formulae for the modes of the feedback system; these showed that the operon architecture leads to slow fixed modes, and suggests a separation principle between the effect of RBS strengths and the promoter characteristic (§3.2).

We also show that engineered pathways consuming an intermediate add further constraints to the RBS strengths design space, which can be relaxed by using promoters with a high dynamic range and small leakiness (§4.1). We performed numerical simulations of the model with physiologically realistic parameters in *Escherichia coli* (§§3.2 and 4.2). The simulations show that the control circuit can effectively upregulate enzyme production to compensate an increase in the cell's native demand for product and the impact of engineered pathways. These also suggest that, in terms of both flux and product homeostasis, the synthetic circuit always outperforms an uncontrolled pathway (i.e. with constant enzyme levels), thus highlighting the advantages of using a dynamic feedback control strategy.

## 2. Unbranched pathway under transcriptional feedback regulation

**Figure 2.**
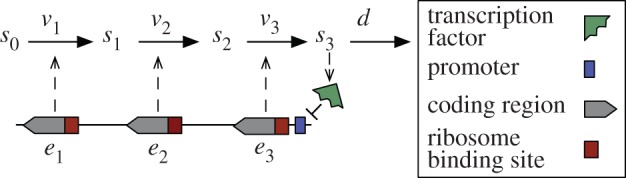
Generic model for an unbranched metabolic pathway under transcriptional repression from the product. The enzymes *e*_*i*_ catalyse the reactions at a rate *v*_*i*_, and the cell consumes the product at a rate *d*. The enzymes are encoded in an operon under the control of a single promoter that is repressed by a TF. (Online version in colour.)

We consider an unbranched metabolic pathway as in figure 2*b*, where *s*_0_ denotes the concentration of substrate, *s*_1_ and *s*_2_ are intermediate metabolites and *s*_3_ is the metabolic product. The metabolic reactions occur at a rate *v*_*i*_ (each one catalysed by an enzyme with concentration *e*_*i*_) and *d* denotes the rate of product consumption by the cell. The metabolic genes are encoded in a single operon controlled by a product-responsive TF that represses enzyme expression. This kind of transcriptional feedback is common, for example, in bacterial nutrient uptake systems (e.g. the lactose operon [21]) and amino acid metabolism (e.g. the tryptophan operon [20]).

### 2.1. Metabolic pathway

The network in figure 2 exchanges mass with the environment and/or other networks in the cell. The model accounts for this interaction via the input substrate *s*_0_ and the product consumption rate *d*. We are interested in biologically meaningful phenotypes, and, therefore, we assume that *s*_0_ is constant to ensure that, the network can reach a non-zero steady state [24]. Note that, if the substrate decays in time, the network eventually reaches a zero equilibrium, whereby the substrate, intermediate metabolites and product are fully depleted. The constant substrate assumption is also suitable for scenarios where *s*_0_ is an extracellular substrate pool shared by a low-density cell population (so that the effects of cell-to-cell competition are negligible).

In a pathway with *n* reactions and *n* metabolites, the rate of change of metabolite concentrations can be described by2.1
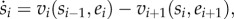
for *i* = 1, 2, … , *n* and *v*_*n*+1_ = *d*(*s*_*n*_). This model arises from the mass balance between the reactions that produce and consume *s*_*i*_, and the enzyme kinetics are included in the reaction rates *v*_*i*_(*s*_*i*−1_, *e*_*i*_). To keep a general analysis, we will not presuppose a specific form for the enzyme kinetics. Instead, we will generically assume that the metabolic reaction rates are linear in the enzyme concentrations [22]2.2
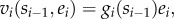
where 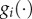 is the enzyme turnover rate (i.e. the reaction rate per unit of enzyme concentration) satisfying *g*_*i*_(0) = 0. We will also assume that the enzyme turnover rates are increasing and saturable functions of the metabolite concentrations, so that2.3
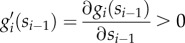
and2.4
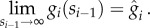
Assumptions (2.2)–(2.4) account for a broad class of saturable enzyme kinetics, including both irreversible Michaelis–Menten and Hill equation kinetics [22,25].

The rate of product consumption *d*(*s*_*n*_) is typically modelled as a saturable function of Michaelis–Menten type [20], but, for the sake of generality, we will consider a generic saturable function *d*(*s*_*n*_) satisfying *d*(0) = 0, 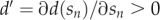 and 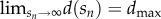 (cf. assumptions (2.3) and (2.4) for the turnover rates). The cellular demand for product depends on the concentration of a product-catalysing enzyme (which is not explicitly modelled in (2.1)). For typical consumption kinetics such as the Michaelis–Menten or Hill equation, the maximal consumption rate *d*_max_ is proportional to the enzyme concentration, and therefore in our model we can describe changes in cellular demand as changes in the parameter *d*_max_.

### 2.2. Synthetic gene circuit

**Figure 3.**
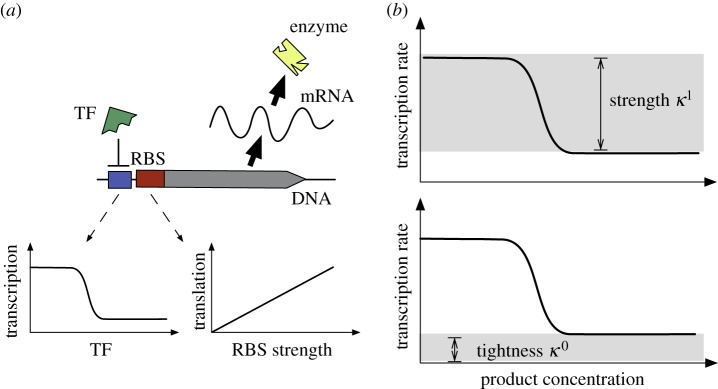
Tuneable knobs in a synthetic operon control circuit. (*a*) The promoter characteristic and RBS strengths modulate gene transcription and translation rates, respectively (the symbols are described in the legend of figure 2). (*b*) Sigmoidal characteristic of a repressible promoter. (Online version in colour.)
  Promoters are typically described in terms of their *tightness* (*κ*^0^) and *strength* (*κ*^1^); see figure 3*b*. The tightness refers to the level of baseline transcription (i.e. under full repression by the product), whereas the strength is the gap between the ON and OFF transcription levels. The promoter strength is quantified in terms of the *dynamic range* *μ*,2.6
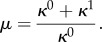
Note that, since the promoter strength is always positive, the dynamic range satisfies *μ* ≥ 1, and we can model the uncontrolled case (i.e. pure constitutive expression without regulation) by taking *μ* = 1.
— *Ribosome binding site strengths*. RBSs are mRNA sequences that are bound by the ribosomes to initiate translation [10]. The translation rate of the enzymes can then be modified by choosing RBS sequences with different affinities to ribosome binding [28]. We model the effect of the RBS strengths on the enzyme expression rate via the parameters *b*_*i*_.
  With the above assumptions and definitions, we can write the complete model for the feedback system as2.7
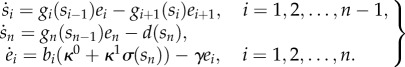
In the remainder of the paper, we focus on the existence and stability of the steady state of the model (2.7), its response to perturbations, and its behaviour as a function of the promoter characteristic and the RBS strengths.

In an operon architecture all the enzymes are under the control of a single promoter (for the multi-promoter case see [26]), and therefore we model the expression of catalytic enzymes as2.5
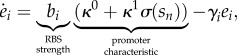
for *i* = 1, 2, …, *n*. This model comes from the balance between protein synthesis and degradation. We consider a first-order degradation process with kinetic constants *γ*_*i*_, which accounts for the aggregate effect of degradation and dilution by cell growth. A common strategy in synthetic biology is to control protein degradation by adding a degradation tag to the gene sequence [27], and thus we assume that all enzymes are tagged and degraded at the same rate, i.e. *γ*_*i*_ = *γ*. In the model (2.5), we have parameterized enzyme expression in terms of the promoter characteristic and RBS strengths, both of which are common design elements in synthetic gene circuits (figure 3*a*):

— *Promoter characteristic*. It describes the regulatory effect of the TF on gene transcription. The function 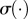 depends on the specific molecular mechanisms underlying the product–TF and TF–promoter interactions. In order to keep a generic description of the regulatory effect, and to parameterize the model in terms of experimentally accessible design parameters, we opt for a phenomenological description of the promoter characteristic. We therefore consider a function 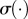 that depends directly on the product concentration and represents the net effect of the product on the transcription rates. Gene transcription under the action of a repressible promoter is typically modelled using Hill functions, but to keep the analysis general we consider generic transcription repression functions satisfying *σ*(0) = 1, 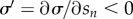 and 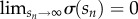.

## 3. Circuit design for cellular demands

### 3.1. Trade-offs and constraints in the design of ribosome binding site strengths

The operon circuit must be able to sustain a metabolic flux that feeds the product into the downstream native processes of the host. In this section, we show how this essential requirement translates into constraints on the RBS strength design space.

We will denote the steady-state metabolite concentrations, enzyme concentrations and reaction rates as 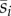, 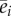 and 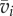, respectively. We first note that the steady-state enzyme concentrations can be obtained by setting 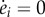 in (2.7), leading to3.1
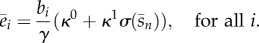
At steady state, the product consumption rate 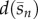 determines the metabolic flux of the network by the relation 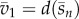, and therefore the steady-state product concentration must satisfy 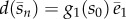. Combining this expression with (3.1) for the first enzyme, we obtain an implicit equation for the steady-state concentration of the product3.2
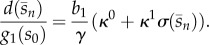


The equilibrium concentrations of the intermediates can be obtained by setting 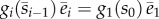 for *i* ≥ 2,3.3
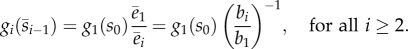
The solution of the implicit equation (3.3) gives the steady-state concentrations of the intermediates as a function of the RBS ratio *b*_*i*_/*b*_1_. Note that because the enzyme turnover rates *g*_*i*_ are monotonically increasing functions, increasing the *b*_*i*_/*b*_1_ ratio leads to a lower steady-state concentration of the intermediate *s*_*i*−1_. From equations (3.2) and (3.3), we can infer how the different tuning knobs affect the steady state of the network:

— *Effect of the promoter characteristic.* From the steady-state equation in (3.2), we can calculate (see appendix A.1 for a detailed derivation) the sensitivity of the product concentration to changes in promoter tightness *κ*^0^, promoter strength *κ*^1^ or RBS strength *b*_1_,3.4
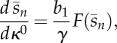
3.5
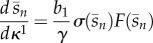
3.6
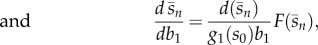
where 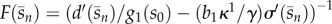. We note that since 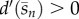 and 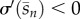, the function 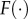 is positive and therefore the sensitivities in (3.4)–(3.6) are also positive. We thus conclude that an increase in the promoter parameters (*κ*^0^ and *κ*^1^) or RBS strength *b*_1_ will lead to a higher steady-state product concentration, which in turn translates into a higher flux. Moreover, since the genes cannot be transcribed at a rate beyond (*κ*^0^+*κ*^1^), from (3.2), we observe that the flux is constrained by the promoter parameters according to3.7
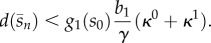
— *Effect of the RBS strengths.* Using (3.1), we can write the steady state of the downstream enzymes as3.8
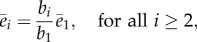
which indicates that a higher *b*_*i*_/*b*_1_ ratio leads to a higher concentration for the *i*th enzyme. Taken together, equations (3.3) and (3.8) indicate that the concentrations of enzymes and intermediates can both be adjusted by tuning the RBS ratio *b*_*i*_/*b*_1_. Comparing the dependencies of (3.3) and (3.8) on the RBS ratio reveals a design trade-off between enzyme expression and the intermediate metabolite concentrations (figure 5*a*): low *b*_*i*_/*b*_1_ ratios lead to low enzyme expression levels at the expense of high concentrations for the intermediates. Conversely, high *b*_*i*_/*b*_1_ ratios tend to increase enzyme expression (and therefore the gene expression burden on the host cell) in favour of low concentrations for the intermediates.

**Figure 4.**
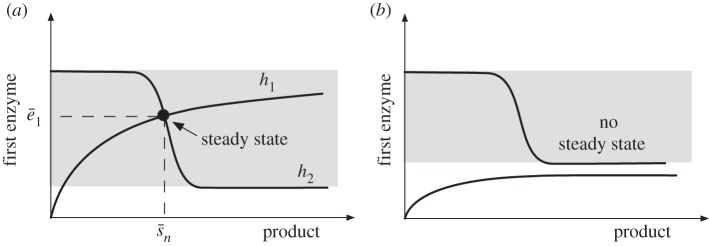
Existence of the metabolic flux, product and first enzyme concentration. (*a*) The solution of steady-state equation (3.2) can be seen as the intersection of two curves, *h*_1_(*x*) = *d(x)*/*g*_1_(*s*_0_) and *h*_2_(*x*) = *b*_1_(*κ*^0^ + *κ*^1^*σ*(*x*))/*γ*. (*b*) The intersection does not exist when condition (3.9) fails.

In the above discussion, we have implicitly assumed that a solution to equations (3.2) and (3.3) exists. However, because of the saturable characteristic of the product consumption rate (*d*) and enzyme kinetics (*g*_*i*_), both equations may lack a solution. Firstly, the solution of (3.2) can be computed as the intersection of the two curves shown in figure 4. From these plots, we can see that an intersection exists only when *d*_max_/*g*_1_(*s*_0_ ) > *b*_1_*κ*^0^/*γ*, or equivalently3.9
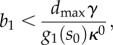
which defines a constraint on the RBS strength of the first enzyme. Since both sides of (3.2) are monotonic in 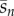, the solution is unique. By equation (3.1), the existence of 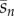 also guarantees the existence of the steady-state enzyme concentrations.

**Figure 5.**
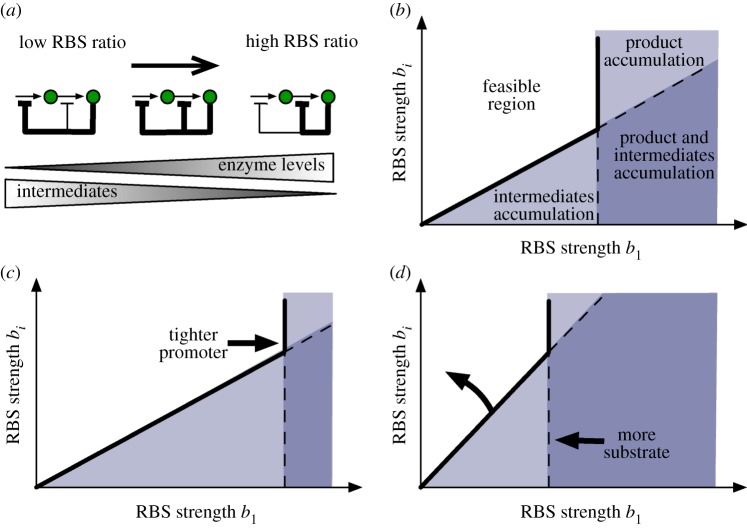
Design trade-offs and constraints for the RBS strengths. (*a*) Design trade-off: a low *b*_*i*_/*b*_1_ ratio yields low steady-state enzyme levels and high steady-state intermediate levels, whereas high ratios increase enzyme concentrations in favour of lower concentrations for the intermediates. (*b*) Design constraint: the feasible RBS region prevents the accumulation of intermediates and product; the region is defined by conditions (3.9) and (3.10). (*c*) Tighter promoters enlarge the feasible region, whereas (*d*) leakier promoters or higher substrate concentrations tighten the constraints. (Online version in colour.)

Secondly, since the enzyme turnover rates saturate at 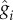, equation (3.3) has a finite solution provided that3.10
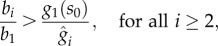
which defines a constraint on the *b*_*i*_/*b*_1_ ratio. Taken together, conditions (3.9) and (3.10) define a feasible region in the RBS strengths design space that prevents the accumulation of intermediates and product (figure 5*b*). If the condition in (3.9) is not satisfied, the substrate will be consumed at a higher rate than the maximal product consumption, and therefore the design will lead to an infinite accumulation of the product. Likewise, violation of at least one of the bounds in (3.10) will cause enzymatic saturation and lead to infinite accumulation of an intermediate.

Conditions (3.9) and (3.10) link together genetic and metabolic parameters (the RBS strengths *b*_*i*_ and promoter tightness *κ*^0^, together with the substrate availability *s*_0_ and the enzyme saturation 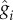), and therefore they shed light on how the design constraints appear due to the interplay between metabolic and enzyme expression dynamics. In figure 5*c*,*d,* we illustrate the effect of promoter tightness and substrate availability on the feasible region for the RBS strengths. Tighter promoters relax condition (3.9) and therefore enlarge the feasible region (figure 5*c*). In the limit case of a perfect leak-less promoter (i.e. *κ*^0^ = 0), condition (3.9) does not limit the RBS strength of the first enzyme. Conversely, by conditions (3.9) and (3.10), a higher substrate tends to tighten the feasible region (figure 5*d*).

### 3.2. Adaptation to changes in cellular demand

One of the purposes of the genetic feedback circuit is to sustain pathway operation under changes in the cellular demand for product. From a control engineering standpoint, a change in cellular demand can be seen as a perturbation signal acting on the network. A useful approach to study dynamical systems under perturbations consists in examining their linear approximation around their equilibrium points. If we write the model (2.7) as 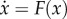 and compute its Jacobian matrix (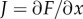), then trajectories starting in a small vicinity of the steady state 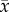 can be approximated as3.11
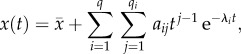
where *λ*_*i*_, *i* = 1, 2, … , *q*, are the *q* distinct eigenvalues of *J* evaluated at 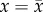, *q*_*i*_ is the algebraic multiplicity of *λ*_*i*_ and the coefficients *a*_*ij*_ depend on the initial conditions and the eigenvectors of the Jacobian. The terms 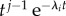 in (3.11), known as feedback modes, provide a local approximation of the trajectories around the equilibrium point.

**Figure 6.**
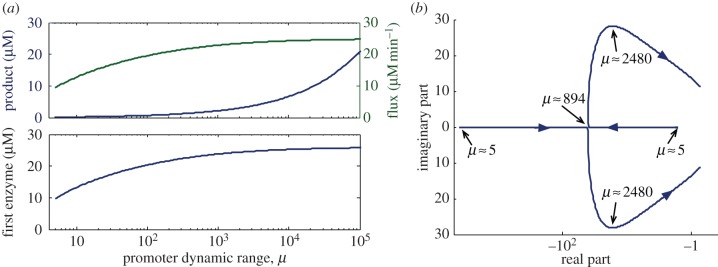
Impact of the promoter dynamic range on the metabolic steady state and the modes of the feedback system. (*a*) Steady-state value of the metabolic flux, and the concentrations of the product and the first enzyme. (*b*) Location of the promoter-dependent eigenvalues *λ*_prom_. The plots were generated by solving the steady-state equation (3.2) (*a*) and computing *λ*_prom_ from (3.13) (*b*) for a different dynamic range *μ* spanning five orders of magnitude and a fixed tightness *κ*^0^. The real and imaginary parts in (*b*) are normalized to the degradation rate *γ*. We considered a pathway with two metabolites and two enzymes with Michaelis–Menten kinetics (*g*_*i*_(*s*_*i*−1_) = *k*_cat *i*_ *s*_*i*−1_/(*K*_M *i*_ + *s*_*i*−1_)) with *k*_cat *i*_ = 32*s*^−1^ and *K*_M *i*_ = 4.7 µM. These are representative values for PRA isomerase (extracted from the BRENDA database [29], EC number 5.3.1.24), a transcriptionally regulated enzyme in the tryptophan pathway of *E. coli*. We took the enzyme degradation rate as 2 × 10^−4^ s^−1^ (half-life 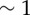 h), and used a substrate concentration *s*_0_ = *K*_M1_ (so that *g*_1_(*s*_0_) is at half-saturation). We modelled the product consumption as a Michaelis–Menten function *d*(*s*_*n*_) = *d*_max_*s*_*n*_/(*K*_d_ + *s*_*n*_) with *d*_max_ = 24.96 µM min^−1^ and *K*_d_ = 0.2 µM, both taken from an experimentally validated model for the tryptophan pathway [20]. Promoter tightness was fixed to *κ*^0^ = 0.03 nM min^−1^ and the RBS strength to *b*_1_ = 1, whereas the repression function was described by a Hill function *σ*(*s*_*n*_) = *θ*^*h*^/(*θ*^*h*^ + *s*_*n*_^*h*^) with Hill coefficient *h* = 2 and repression threshold *θ* = *K*_d_. (Online version in colour.)

In the case of the feedback system in (2.7), we can exploit the structure of the Jacobian matrix to obtain analytic expressions for its eigenvalues in terms of the design knobs of the gene circuit (see appendix A.2 for details). We found that the 2*n* eigenvalues can be classified into three categories *λ*_fixed_, *λ*_RBS*i*_ and *λ*_prom_. The system has the following:

— (*n*−1) stable eigenvalues at *λ*_fixed_ = −*γ* < 0. These eigenvalues are independent of the circuit design parameters, and therefore they lead to *fixed modes,* which can be adjusted only by changing the degradation rate (e.g. with various degradation tags). They cannot be suppressed or changed by tuning the circuit design knobs, and, from (3.11), we see that they translate into (*n* − 1) modes of the form e^−*t*/*γ*^, *t* e^−*t*/*γ*^, … , *t*^*n*−2^ e^−*t*/*γ*^. The enzyme degradation rates *γ* are inversely proportional to their half-lives, which are in turn much longer than metabolic time scales (enzymatic half-lives are of the order of minutes to hours, whereas metabolic time scales are typically milliseconds to seconds [22]). Therefore, depending on the initial conditions the network can potentially display very slow transients, and this appears to be aggravated in long pathways.
— (*n*−1) stable eigenvalues at3.12
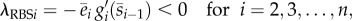
with 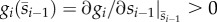. Since the steady-state concentration of the enzyme 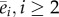, and the intermediate 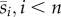, depend only on the corresponding RBS ratio *b*_*i*_/*b*_1_ (see equations (3.3) and (3.8)), this ratio can be used to independently fine-tune the feedback mode associated with *λ*_RBS*i*_.
— Two stable eigenvalues at3.13
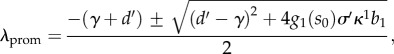
with 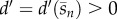 and 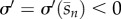. Unlike *λ*_fixed_ and *λ*_RBS*i*_, these two eigenvalues depend on the steady-state product concentration, and therefore they can be fine-tuned through the promoter characteristic (see equation (3.2)). To study the dependence of *λ*_prom_ on the promoter design parameters, we computed them for a pathway with realistic parameter values. In figure 6*a,* we show the steady-state values of the product, flux and first enzyme level for a wide span of promoter dynamic range *μ*. We observe that strong promoters tend to increase pathway flux, in agreement with the sensitivity equation previously derived in (3.5). We also see that, as shown by the steady-state relation 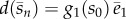, the flux corresponds to a scaled version of the concentration of the first enzyme. In figure 6*b,* we plot the location of the promoter-dependent eigenvalues *λ*_prom_ in the complex plane. These indicate that, in the case of weak promoters, the eigenvalues *λ*_prom_ lie on the real axis, becoming complex only for a sufficiently broad dynamic range *μ*. For strong promoters, the real part 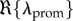 becomes closer to the imaginary axis, potentially leading to slow transients. Moreover, since stronger promoters lead to a higher flux, the eigenvalues in figure 6*b* suggest that flux maximization may entail a reduction in the response speed.

**Figure 7.**
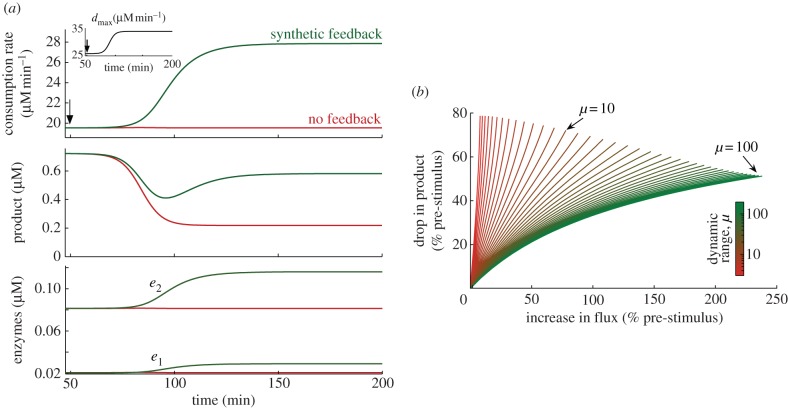
Response of a synthetic operon to changes in the cellular demand for product. (*a*) Comparison between the dynamic responses of the synthetic control circuit and the uncontrolled case; the change in cell demand was induced at *t* = 50 min and modelled as an S-shaped increase in the maximal consumption rate *d*_max_, reaching 50% of the pre-stimulus *d*_max_ in 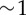 h (see inset). (*b*) Drop in the steady-state product concentration relative to its pre-stimulus value as a function of the change in metabolic flux and the promoter dynamic range. In (*a*), the promoter dynamic range was set to *μ* = 100 and the RBS strengths to *b*_*i*_ = {1,4}; for the pathway parameters used in figure 6, the chosen *b*_*i*_ comply with the feasible region of figure 5 and lead to steady-state enzyme concentrations that are within the physiological range of *E. coli* [30]. In (*b*), we swept the dynamic range *μ* between 3 and 200 by changing the promoter strength. All the remaining network parameters are the same as in figure 6. (Online version in colour.)

To illustrate the dynamic response of a pathway under the control of the transcriptional control circuit, we simulated the network under a change in the cell demand for product (see figure 7*a*). We modelled a change in the cell demand as a slow S-shaped temporal increase in the maximal product consumption rate *d*_max_ (see the inset in figure 7*a*). This describes, for example, cases in which the demand increase is due to native processes upregulating the enzyme that metabolizes the product. Note that, from the steady-state equation in (3.2), a higher *d*_max_ inevitably leads to a higher flux and a lower steady-state product concentration (see also figure 4*a*). Before the perturbation, the network is in steady state with a pre-stimulus flux *d*^pre^ = 19.5 µM min^−1^. We considered a Michaelis–Menten consumption rate of the form *d*(*s*_*n*_) = *d*_max_*s*_*n*_/(*K*_d_ + *s*_*n*_), with *K*_d_ being the product concentration needed for half-maximal consumption. Upon the increase in *d*_max_ at *t* = 50 min, we observe that the promoter responds to the drop in product concentration and upregulates enzyme expression so as to drive the pathway to a new post-stimulus flux *d*^post^ that is approximately 40 per cent higher than *d*^pre^, and a product concentration that is approximately 20 per cent lower than its pre-stimulus value. Using equations (3.1) and (3.2), we can compute the enzyme upregulation factor as3.14
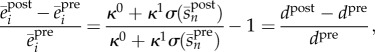
which is equivalent to the relative change in pathway flux. The dynamic upregulation of enzyme expression can be seen in the lower panel of figure 7*a*, where we can also verify that the upregulation factor is approximately 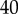 per cent as predicted in (3.14). Note that as a consequence of the operon architecture, all the enzymes are upregulated by the same fold-factor. This factor depends on the pre- and post-stimulus fluxes, which by (3.2) depend only on the promoter design and first RBS strength.

As a way of comparison, in figure 7*a,* we have also simulated the response of a pathway without feedback regulation (i.e. with constant enzyme levels 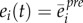 chosen to match the flux of the controlled case *d*_pre_). The uncontrolled pathway is unable to increase the flux, and we observe that this leads to a considerable decrease in product concentration (approx. 70% reduction). In the uncontrolled case, the rate of substrate uptake is fixed to *v*_1_^unc^ so that the equilibrium product satisfies3.15
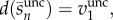
and therefore any increase in *d*_max_ translates into a lower product concentration 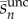, which in turn depends only on the kinetic parameters of the consumption rate *d*(*s*_*n*_). In contrast, the feedback-controlled pathway can partly compensate the drop in product by dynamically upregulating enzyme expression, substantially outperforming the uncontrolled case.

We study the performance of the control circuit in more detail in figure 7*b*, where we show the drop in product concentration relative to the pre-stimulus level as a function of the change in flux and dynamic range of the promoter. We observe that stronger promoters can significantly improve the compensation of the drop in product concentration (a perfect compensation would correspond to a flat curve at 0% in figure 7*b*). For example, under a 50 per cent increase in the pathway flux, a mild promoter (*μ* = 10) leads to a drop in product of approximately 47 per cent, whereas a strong promoter (*μ* = 100) can bring down the drop in product to approximately 20 per cent (the latter corresponds to the design simulated in figure 7*a*). As predicted by the upper bound in (3.7), the flux is limited by the promoter strength, and therefore weak promoters do not allow for large increases in flux (as a consequence, the domain of the curves in figure 7*a* decreases with decreasing promoter strength); for example, for the weakest promoter tested, the flux could not be increased beyond approximately 10 per cent.

## 4. Circuit design for compensation of flux perturbations

A common strategy in metabolic engineering is to modify bacteria by expressing heterologous enzymes that convert natural metabolic intermediates into a compound of interest [19]. The target compound is synthesized by ‘branching out’ a specific intermediate from a natural pathway, and therefore part of the metabolic flux needed to sustain the host native processes is redirected to the production of the foreign chemical. The choice of a good branching point (i.e. one that does not lead to lethal metabolic imbalances for the host) is a major problem typically addressed with the aid of optimization-based computational tools [31,32]. In this section, we turn our attention to the effect of a perturbation in the native flux as a consequence of branching out from an intermediate metabolite.

### 4.1. Trade-offs and constraints in the design of the RBS strengths

**Figure 8.**
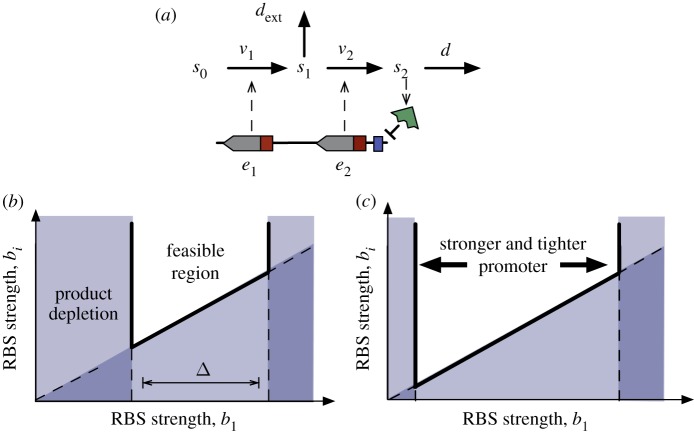
Design constraints of the RBS strengths with a branch consuming an intermediate. (*a*) Metabolic pathway under transcriptional regulation with a branch consuming an intermediate. Model equations are shown in (4.1) and symbols are described in the legend of figure 2. (*b*) In addition to the constraints in figure 5*b*, the branch introduces a further constraint to avoid product depletion; the region is defined by conditions (4.5)–(4.7). (*c*) Stronger and tighter promoters enlarge the gap *Δ* and the RBS design space according to equation (4.8). (Online version in colour.)

To account for an engineered pathway consuming the intermediate 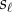 at a constant rate *d*_ext_, we include *d*_ext_ as a consumption rate in the ODE for 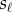
4.1
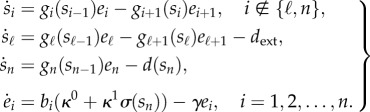
The system is shown in figure 8*a*, and in this case the steady-state equation for the product and the first enzyme is4.2
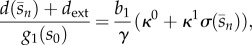
where the left-hand side is a shifted version of the one in (3.2). For the intermediates before the branch point, the steady-state concentration is given by the same equation as in (3.3)4.3
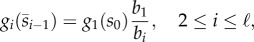
whereas for the intermediates after the branch point, we have a modified equation4.4
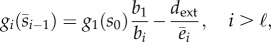
with 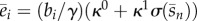. From these steady-state equations, we observe similar properties as in the case without a branch. The promoter characteristic and the first RBS strength determine the metabolic flux, whereas the RBS ratio *b*_*i*_/*b*_1_ can be used to fine-tune the balance between enzyme expression and the concentrations of the intermediates. In addition, in this case, we see that the intermediates after the branch point also depend on the promoter characteristic.

Using similar arguments to those in figure 4, we find that a solution to (4.2) exists if4.5
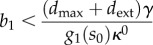
and4.6
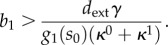
For equation (4.4) to have a solution, in principle, we need 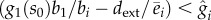, but this condition is less stringent than the one previously derived in (3.10) for the case *d*_ext_ = 0. Since the design must also prevent the accumulation of intermediates in the absence of perturbations, we conclude that (3.10), i.e.4.7
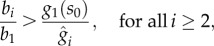
is sufficient for the existence of all the intermediates. The inequalities in (4.5)–(4.7) define the feasible region for the RBS design space under a perturbation consuming one of the intermediates (see figure 8*b*). As in the case without the branch (figure 5*b*), the limits (4.5) and (4.7) prevent the accumulation of the product and intermediates, respectively. The condition in (4.6), however, adds a new type of constraint to the design space: it guarantees that the synthetic gene circuit can upregulate enzyme expression strongly enough to cope with the flux through the branch, hence preventing the depletion of the product. This new constraint also depends on the promoter dynamic range, which was absent in the case without a branch. From (4.5) and (4.6), we can compute the gap between the upper and lower bounds for the first RBS strength (see figure 8*b*)4.8
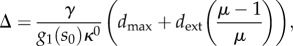
which reveals that promoters with a broad dynamic range and small leakage enlarge the RBS design space (see figure 8*c*).

### 4.2. Adaptation to a flux perturbation

**Figure 9.**
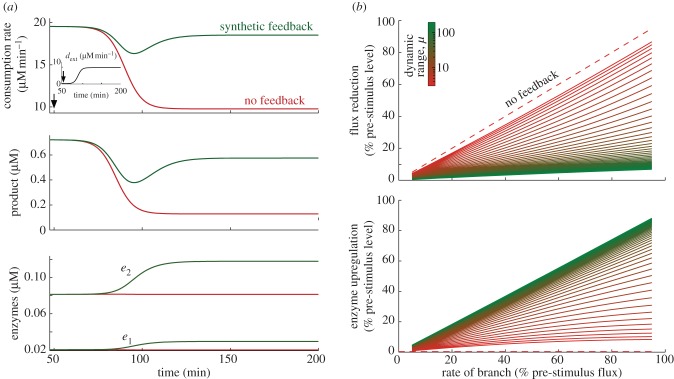
Response of a synthetic operon to flux perturbations induced by engineered pathways. (*a*) Comparison between the dynamic responses of the synthetic control circuit and the uncontrolled case; the flux perturbation was induced at *t* = 50 min, and modelled as an S-shaped increasing rate consuming the intermediate *s*_1_, reaching 50% of the pre-stimulus flux in 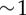 h (see the inset). (*b*) Flux reduction and enzyme upregulation relative to the pre-stimulus levels as a function of the consumption rate in the branch and the promoter dynamic range. The uncontrolled case (*μ* = 1) is represented by the dashed lines. As in figure 7, in (*a*) we chose a promoter with dynamic range *μ* = 100 and RBS strengths *b*_*i*_ = {1,4}, whereas in (*b*) we swept *μ* between 3 and 200 by changing the promoter strength. All the remaining network parameters are the same as in figure 6. (Online version in colour.)

To illustrate the effect of an engineered branch on the dynamic response of the feedback system, we simulated a network with two metabolites and two enzymes under a flux perturbation that consumes the intermediate *s*_1_ (see figure 9*a*). Before the perturbation, the network is in steady state with a native flux *d*^pre^ = 19.5 µM min^−1^. We modelled the engineered branch as an S-shaped increasing rate *d*^ext^(*t*) (see the inset in figure 9*a*). Upon the activation of the branch, induced at *t* = 50 min, the synthetic operon circuit upregulates enzyme expression by approximately 45 per cent to drive the pathway to a new native flux *d*^post^. Using equation (3.1), together with the pre- and post-stimulus steady-state equations ((3.2) and (4.2)), we find that the enzymes are upregulated by the factor4.9
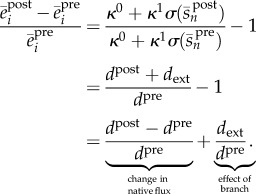

As in the case without the branch, the expression in (4.9) indicates that all enzymes are upregulated by an identical fold-factor that depends on the promoter design and the first RBS strength.

In figure 9*a,* we have also simulated the response of a pathway without feedback regulation (i.e. with constant enzyme levels 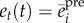 chosen to match the flux *d*^pre^ of the controlled case). In terms of both flux and product concentration, we observe that the feedback-controlled network displays a dramatic improvement compared with the uncontrolled case: the operon circuit reduces the loss in native flux from 50 per cent to approximately 5 per cent, whereas the decrease in steady-state product concentration is brought down from approximately 82 per cent to 20 per cent. In the uncontrolled case, the rate of substrate uptake is fixed to *v*_1_^unc^ and therefore the post-stimulus flux is given by4.10
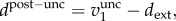
and hence the post-stimulus flux scales linearly with the rate of the branch. In figure 9*b,* we show this linear dependence together with the feedback-regulated case for a wide span of the promoter dynamic range. We observe that the feedback control circuit outperforms the uncontrolled case even with promoters with a narrow dynamic range, and that this improvement can be achieved with a relatively low enzyme upregulation factor.

## 5. Discussion and outlook

In this paper we have presented a detailed analysis of a synthetic gene circuit designed to dynamically control metabolic pathways. The goal of this feedback control system is twofold: to adjust pathway activity so as to match the cell demand for product, and to dampen flux perturbations that divert the native flux to the synthesis of foreign molecules. The control strategy relies on encoding the metabolic genes in a single operon repressed by a product-responsive TF. The TF can sense a drop in product concentration and upregulate enzyme expression to bring the pathway close to its homeostatic levels.

Since the seminal operon paper [33], the interaction between the genetic machinery and metabolism has been extensively studied in the context of natural systems. These studies typically focus on understanding how observed phenotypes emerge from the genetic–metabolic cross talk [34–38], and a number of detailed mechanistic models for operon regulation have been developed (e.g. [20,21]). The goal in synthetic biology, however, is to design regulatory circuits for controlling metabolism in a customized fashion. Model-based design therefore requires mathematical descriptions that are explicitly parameterized in terms of the design knobs that can be manipulated in synthetic biology applications. Consequently, we have used a gene expression model that is deliberately not mechanistic, and instead describes the genetic feedback in terms of tuneable parameters such as the promoter's dynamic range, RBS strengths and protein half-lives. This approach has proved to be adequate to explore the genetic design space and to quantify the impact of the promoter characteristic and RBS strengths on the system response.

A typical complication in engineered pathways is that enzymatic saturation may cause intermediates to accumulate in prohibitively large concentrations, thus affecting the viability of the host due to toxic effects [11]. Metabolite accumulation arises when the steady state lies beyond the saturation limit of a catalytic step, and available models for pathways under transcriptional regulation [20,39–41] have generally overlooked the impact of enzyme saturation on the existence of a metabolic steady state. In our aim to carry out a general analysis, we have used a metabolic model that accounts for a whole class of saturable enzyme kinetics under mild assumptions. By explicitly accounting for enzyme saturation, we characterized a feasible set for the design parameters which ensures that the steady state lies within the saturation limits. The feasible set also guarantees the local stability of the network, and we found that the constraints on the RBS strengths can be relaxed with the use of promoters with a high dynamic range and small leakiness. The geometry of the feasible set depends on a combination of genetic and kinetic parameters, thus highlighting the emergence of design constraints as a consequence of the interplay between the genetic and metabolic subsystems.

The steady-state equations reveal a trade-off between the steady-state enzyme expression levels and the concentration of intermediates: the enzyme concentrations are inversely proportional to the concentration of the intermediate they catalyse. We found that a critical parameter is the RBS ratio, i.e. the relative strength of an RBS with respect to the strength of the first one in the operon, which can be used to fine-tune the circuit between high-enzyme/low-intermediate or low-enzyme/high-intermediate designs.

The two considered design knobs, promoter characteristic and RBS strengths, seem to have decoupled roles in the steady state and transient behaviour of the network. The promoter characteristic together with the first RBS determine the steady state of the product and the first enzyme. A strong promoter and a strong RBS for the first enzyme can be used to increase the pathway flux, but this may come at the expense of slow modes in the transient response. In the absence of an engineered pathway consuming an intermediate, the remaining RBS strengths can be used to independently adjust the concentrations of the intermediates and the remaining enzymes. In the case of consumption of an intermediate, however, this design rule applies only to the metabolites upstream of the consumed intermediate, i.e. the steady state of the downstream metabolites depends on a combination of the RBS strength, promoter characteristic and the size of the perturbation.

The closed-form expressions for the transient modes of the feedback system show further evidence of the separation principle between promoter and RBS design. From the 2*n* modes of an *n*-step pathway, we found that only two depend on the promoter characteristic, whereas further (*n* − 1) modes depend exclusively on the RBS ratio. The remaining (*n* − 1) modes correspond to the enzyme half-lives and are independent of the promoter characteristic and RBS strengths. Since enzyme half-lives are considerably slower than metabolic time constants (even with the use of protein degradation tags), the system dynamics can be dominated by slow transients.

We ran numerical simulations that demonstrate the potential of the proposed control strategy. Using physiologically realistic parameter values for *E. coli*, the synthetic operon control circuit can dramatically compensate the loss in flux by sensing the drop in product concentration and subsequently upregulating the enzyme concentrations. The feedback-controlled pathway outperforms the uncontrolled one even when weak promoters are used, thus underscoring the tremendous advantage of taking a feedback approach to metabolic control.

In this work we focused on a control circuit with an operon architecture, a choice inspired by the fact that operons are one of the building blocks in genome-wide bacterial networks [42]. The ubiquity of natural metabolic pathways under operon regulation [43] makes them a reasonable choice as template architectures for engineered circuits. In addition, the main difficulty in building genetic–metabolic systems is to find suitable regulatory molecules to interface a metabolite of interest with the genetic machinery. Some of the available alternatives are engineered promoters [44,45], metabolite-responsive riboswitches [18,46,47] and natural TFs (for a comprehensive catalogue of natural metabolite-responsive TFs see Zhang *et al*. [14, supp. table 5]). In this respect, an operon architecture stands as a simple yet effective topology, as it requires only one metabolite-responsive TF. More complex architectures can certainly add more flexibility to the design, but this will probably come at the expense of more intricate relationships between the design parameters and the metabolic response. For example, the use of multi-promoter circuits allows for independent tuning of the enzyme upregulation factor, but at the same time the pathway may display sustained oscillations if the characteristics of the different promoters are not carefully designed [26].

We should point out that the derived design constraints guarantee the existence and stability of the metabolic steady state, and thus they are only baselines for the correct functioning of the genetic control circuit. In most applications, the design must also account for more demanding objectives such as maximization of flux, minimization of energy expenditure, or a combination of these. Since these objectives may conflict with each other, selecting an appropriate combination of circuit parameters requires the use of multi-objective optimization methods within the feasibility sets derived here (see, for example, figures 5 and 8). Optimization routines can therefore be used to single out the parameter values that lead to an acceptable compromise between mutually colliding objectives; see Banga [48] for a review of a number optimization methods available.

As a consequence of a compromise between model complexity and the generality of the analytic results, our results have two main limitations. Firstly, we have restricted the analysis to pathways with irreversible reactions, and, secondly, our results are limited to unbranched pathways operating in isolation of the remaining metabolism of the host cell. Enzymatic reactions are inherently reversible processes and, although many biosynthetic reactions operate in a regime where the forward reaction is much more likely to occur than its backward counterpart [22], their reversibility cannot always be neglected [49]. In our case, the use of irreversible reactions is an important simplification that allowed the derivation of intuitive and easy to interpret relations between the network parameters and its steady state. Other instances where the analysis of irreversible pathways led to new insights into biological design principles include, for example, the works in [38,43]. Our derivation of the design constraints on the promoter parameters and RBS strengths relies on the structure of the steady-state equations and the fact that most of them are decoupled from each other. However, in the case of an *n*-step pathway with reversible reactions, the steady-state equations form a system of 2*n* coupled algebraic equations. These equations may admit an analytic solution for specific enzyme kinetics (see Heinrich & Klipp [50] for the solution in the case of linear and Michaelis–Menten kinetics with constant enzyme concentrations), but its extension to transcriptionally controlled enzymes and general reversible kinetics is cumbersome and lies outside the scope of our paper.

A possible workaround to deal with reversible kinetics is to exploit the natural timescale separation between enzyme expression and metabolic reactions. In this approach, the metabolite trajectories are assumed to evolve in a much faster time scale than the enzyme concentrations. This allows us to approximate the metabolite concentrations as algebraic functions of the enzymes, leading to an enzyme-only ODE model subject to the algebraic relations between metabolites and enzymes. We have previously used such an approach in the case of ON–OFF promoters [36] (i.e. promoters that are either fully active or inactive, without intermediate levels of gene expression), and future work will focus on its use with graded promoters such as those considered here. Another advantage of the time scale separation is that it may allow for the analysis of pathways with more complex stoichiometries. This is of enormous relevance in practical applications, as the cross talk between the controlled pathway and the rest of the host metabolism is likely to have a detrimental impact on the performance of the feedback control system.

We are exploring a number of extensions to this work, aiming primarily at the use of alternative feedback topologies and at quantifying the impact of biochemical noise on the pathway performance. The implementation of genetic–metabolic circuits, let alone parameter fine-tuning, can be costly and time-consuming. Our work provides a first step towards understanding the fundamental limitations and trade-offs that must be addressed at the design stage, potentially facilitating the implementation using a model-guided rationale.

## Appendix A

**A.1. Derivation of the sensitivities**

To obtain the sensitivities in (3.4)–(3.6), we differentiate the steady-state equation in (3.2) with respect to the parameter of interest, and then use the chain rule. For example, to obtain (3.4) we differentiate (3.2) with respect to *κ*^0^

A1

and then solve for  to obtain

A2

where . The remaining sensitivities  and  can be calculated in an analogous manner.

**A.2. Local stability analysis**

Here we show that, under the conditions (3.9) and (3.10), the network (2.7) has a locally stable steady state. Moreover, its Jacobian has:

— *n*−1 stable eigenvalues at *λ*_fixed_ =−*γ*;
— *n*−1 stable eigenvalues at , ; and
— and two stable eigenvalues at

A3

We first write the vector of metabolite and enzyme concentrations as *s* and *e*, respectively, so that the model (2.7) can be written as

A4

The conditions in (3.9) and (3.10) guarantee the existence of the steady state. The entries of the Jacobian matrix are

A5

A6

A7

A8

and the coefficients *a*_*i*_ are defined as

A9

and

A10

Note that because *g*_*i*_ and *d* are non-decreasing, it follows that *a*_*i*_ ≥ 0. Using (3.3), we can write the terms  in terms of the RBS ratio

A11

We can therefore write the Jacobian as a block matrix

A12

where the four blocks are  matrices

A13

A14

A15

The characteristic polynomial of *J* (i.e. *p*(*λ*) = det( *J* − *λ**I*)) is

A16

where the product *J*_12_*J*_21_ is

A17

and *q* = *g*_1_(*s*_0_ )*σ*′*κ*^1^*b*_1_. From the structure of *J*_11_ and the product *J*_12_*J*_21_, we can carry one (*λ* + *γ*) term into the determinant in (A 16) and then into the last column of its argument. This leads to

A18

The factor  is a polynomial given by

A19

We therefore conclude that the Jacobian *J* has (*n*−1) stable eigenvalues at *λ*=−*γ* < 0, (*n*−1) stable eigenvalues at *λ*=−*a*_*i*_ < 0, for *i* = 2, 3, …, *n*, and two eigenvalues at

A20

These last two eigenvalues are also stable because  and  imply that the quadratic polynomial

A21

has only positive coefficients and therefore its roots satisfy .
